# Polymorphisms in vasoactive eicosanoid genes of kidney donors affect biopsy scores and clinical outcomes in renal transplantation

**DOI:** 10.1371/journal.pone.0224129

**Published:** 2019-10-17

**Authors:** Sonia Mota-Zamorano, Luz M. González, Enrique Luna, José J. Fernández, Áurea Gómez, Alberto Nieto-Fernández, Nicolás R. Robles, Guillermo Gervasini

**Affiliations:** 1 Department of Medical and Surgical Therapeutics, Division of Pharmacology, Medical School, University of Extremadura, Badajoz, Spain; 2 Service of Nephrology, Badajoz University Hospital, Badajoz, Spain; 3 Service of Anatomical Pathology, Infanta Cristina University Hospital, Badajoz, Spain; Max Delbrueck Center for Molecular Medicine, GERMANY

## Abstract

Cytochrome P450 (CYP) enzymes metabolize arachidonic acid to vasoactive eicosanoids such as epoxyeicosatrienoic acids (EETs) and 20-Hydroxyeicosatetraenoic acid (20-HETE), whilst soluble epoxide hydrolase, encoded by the *EPHX2* gene, is in charge of EETs degradation. We aimed to analyze the influence of common, functional polymorphisms in four genes of the donor on the renal biopsy scores independently assigned by pathologists. Additionally, we examined whether this score or the presence of these SNPs were independent risk factors of clinical outcomes in the first year after grafting. A cohort of 119 recipients and their corresponding 85 deceased donors were included in the study. Donors were genotyped for the *CYP4F2 V433M*, CYP2C8*3, CYP2J2*7, *EPHX2* 3’UTR A>G, *EPHX2* K55R and *EPHX2* R287Q polymorphisms. The association of the donors’ SNPs with the biopsy scores and clinical outcomes was retrospectively evaluated by multivariate regression analysis. The CYP2C8*3 polymorphism in the donor was significantly associated with higher scores assigned to pretransplant biopsies [OR = 3.35 (1.03–10.93), p = 0.045]. In turn, higher scores were related to an increased risk of acute rejection [OR = 5.28 (1.32–21.13), p = 0.019] and worse glomerular filtration rate (eGFR) (45.68±16.05 vs. 53.04±16.93 ml/min in patients whose grafts had lower scores, p = 0.010) one year after transplant. Patients whose donors carried the *CYP4F2* 433M variant showed lower eGFR values (48.96±16.89 vs. 55.94±18.62 ml/min in non-carriers, p = 0.038) and higher risk of acute rejection [OR = 6.18 (1.03–37.21), p = 0.047]. The CYP2J2*7 SNP in the donor was associated with elevated risk of delayed graft function [OR = 25.68 (1.52–43.53), p = 0.025]. Our results taken together suggest that donor genetic variability may be used as a predictor of tissue damage in the graft as well as to predict clinical outcomes and graft function in the recipient.

## Introduction

Chronic kidney disease is a public health problem characterized by the progressive loss of renal function that affects an important part of the world’s population. The patients in the most advanced state of the disease are diagnosed with end-stage renal disease (ESRD) and they need either dialysis treatment or a kidney transplant. The latter is the first-choice treatment but the unavailability of adequate organs for transplantation to meet the existing high demand has resulted in a severe shortage of deceased donor organs [1].

It has been suggested that in many cases the decision to refuse marginal donor kidneys could be unjustified. There is a need for more rigorous and standardized criteria to accept or reject the organ, in order to minimize the discard of transplantable kidneys [1, 2]. For this reason, there exist different histopathological scoring systems to assess the donor’s kidney status [3, 4]. This, together with clinical parameters, help clinicians predict graft dysfunction or loss.

There is an increasing body of research highlighting the importance of the arachidonic acid (AA) epoxygenase route in renal transplantation [5, 6]. In this pathway, the cytochrome (CYP) P450 metabolizes AA locally in the kidney into vasoactive eicosanoids such as epoxyeicosatrienoic acids (EETs), which display renoprotective properties [7, 8] or hydroxyeicosatetraenoic acids, especially 20-HETE, with a myriad of vascular functions and that has even been proposed as a biomarker of post-transplant allograft function [9]. EETs are biotransformed to less active dihydroxyeicosatrienoic acids (DHETEs) by soluble epoxide hydrolase, encoded by the *EPHX2* gene. Therefore, single nucleotide polymorphisms (SNPs) occurring in this pathway hold the potential to affect graft function. Indeed, we have previously shown that the presence of these genetic variants in the donor’s DNA is associated with delayed graft function (DGF) [10], acute rejection [11, 12] or creatinine clearance [10]. Amongst the most studied SNPs in CYP genes of this route are CYP2C8*3 and CYP2J2*7, both of which have been associated with decreased enzymatic activity or lower transcription rate in several in vitro studies [13–15]. In addition, *CYP4F2* V433M has also been claimed to be a loss-of-function SNP in vitro [16], although there exists some controversy in this regard [17]. Finally, with regard to the *EPHX2* gene, R287Q has been related to decreased enzyme function [18], whilst K55R and a A/G transition in the 3’UTR are believed to increase the activity or expression of sEH [18, 19]. We aim to examine whether these functional, common polymorphisms may be associated with the scores assigned by pathologists to kidney biopsies and, in turn, related to an unfavorable evolution of the graft.

## Subjects and methods

### Study design

The study group consisted of 119 renal transplant recipients and their respective 85 deceased donors (in several cases both kidneys from one donor were transplanted into different recipients). Both donors and recipients were all of Caucasian origin. After the transplant, which were carried out at the Badajoz University Hospital (Spain), all patients received triple immunosuppressive therapy with either tacrolimus or cyclosporine, 2 g/day mycophenolate mofetil and a tapering schedule of corticosteroids. Tacrolimus and cyclosporine blood concentrations were routinely measured using an immunoassay performed on a Cobas Mira Plus analyzer (Roche Diagnostics).

### Ethics statement

Participants in the study were recruited on the day of their scheduled visit to the Renal Transplant Follow-up Unit, where they gave verbal and written informed consent for their participation. The study was approved by the Bioethics Committee of the Badajoz University Hospital and was conducted in accordance with the Declaration of Helsinki and its subsequent revisions. No minors under age 18 were included in the study. None of the transplant donors were from a vulnerable population and all donors or next of kin provided written informed consent that was freely given.

### Assessment of renal biopsies and clinical variables

Clinicians of the Service of Pathological Anatomy, who were blind to genotype, performed the evaluation of the pretransplant kidney biopsies according to a consensus document previously published by Spanish nephrologists [20]. Briefly, five items were considered for the calculation of the biopsy score: glomerular sclerosis, hyaline arteriopathy, thickening of vascular intima, tubular atrophy and interstitial fibrosis (these two last items were jointly evaluated). The assessment of these parameters was made in a semiquantitative scale from 0 to 3 according to the Banff criteria [21]. A total score of less than 5 was indicative of minor damage, 5–6 indicated mild damage and over 6 designated severe damage.

Delayed graft function (DGF) was defined as the need for dialysis within the first week after transplantation [22]. Acute allograft rejection was established by histological findings in renal biopsies of recipients according to the Banff classification and/or by clinical evaluation as previously described [5, 23]. Renal function was measured by the estimation of eGFR from serum creatinine with the Modification of Diet in Renal Disease (MDRD) formula.

### Genotype analysis

Genomic DNA was isolated from frozen lymphocytes obtained from the deceased donors by using a QIAamp DNA Blood Kit (Qiagen, Hilden, Germany). Six common, functional SNPs in four genes of the epoxygenase pathway were examined, namely CYP2C8*3 (rs10509681), CYP2J2*7 (rs890293), *CYP4F2* V433M (rs2108622) and *EPHX2* 3’UTR A>G, K55R (rs41507953) and R287Q (rs751141). Genetic variants were identified by RT-PCR techniques using commercially available Taqman^®^ probes from Life Technologies (Maryland, USA). These polymorphisms were selected on the basis of (i) a reported effect on EETs or 20-HETE levels [13, 16, 24] and/or (ii) because of their impact on the evolution of the graft [5, 10, 11].

### Statistical analyses

Fisher’s exact or Pearson’s X^2^ test were used for the univariate analysis of the associations between categorical data. In order to compare mean values of quantitative variables between different groups, we used t-Student’s/ANOVA or Mann-Whitney/Kruskal-Wallis tests, as appropriate. Multivariate regression analyses were performed in order to evaluate the influence of genetic and non-genetic covariates, which were included according to statistical significance observed in univariate analyses and/or clinical criteria. The covariates finally included in each analysis are either specified in the text or listed in the tables depicting the resulting models. The complete set of covariables from both donors and recipients that were considered in the study is shown in supplementary S1 Table. Biopsyscores were transformed into a binary variable for the association analyses, therefore generating a low-score group for scores 1 to 4 and a high-score group for 5 to 8. All datasets are available upon request.

In the absence of previous reports indicating a clear gene-dose effect for the studied polymorphisms, a dominant model of inheritance, i.e. carriers vs. non-carriers, was used to perform genetic association analyses. This approach was selected based on our previously reported findings in kidney transplantation [5, 10–12, 25, 26] and also with the intention of balancing the size of both study groups.

In order to determine the weight of the donor genetics compared to recipients characteristics with regard to their influence on clinical outcomes, we analyzed a subgroup of 68 kidneys that came from 34 donors, i.e. kidney pairs that had the same genetic background. Paired t-tests were utilized to determine putative differences between paired organs.

The statistical power of the study was evaluated with a genetic model established analysing the frequency for carriers of the variant alleles with an arbitrarily effect size set at 2.5 (type I error = 0.05). With the available sample size, the power for detecting genotype-phenotype associations ranged from 0.77 to 0.82 depending on the minor allele frequency and the reported incidence of the measured outcome (Quanto Software v. 1.2.4, USC).

Statistical analyses were performed with the IBM SPSS statistics 22 (Chicago, IL) and the *SNPassoc* R package [27]. This software is available at https://cran.r-project.org/web/packages/SNPassoc/index.html and can be added to the R environment to obtain descriptive statistics and exploratory analysis of missing values, calculation of Hardy-Weinberg equilibrium and analysis of genetic associations based on generalized linear models (either for quantitative or binary traits).

## Results

A total of 119 renal transplant recipients (78 men and 41 women) with a mean age of 57.34 ± 10.37 years were included in the study. They received kidneys from 85 deceased donors. The cause of death was head trauma (11.4%), stroke (79.5%) or it was unavailable (9.1%). The mean age of the donors, 54 of whom were males, was 61.65 ± 9.24 years. In the first year after grafting there were 43 cases of delayed graft function (36.1%) and 18 of acute rejection (15.1%) among the renal transplant recipients. Twenty seven patients experienced graft loss (22.7%). These and other clinical and demographic data are depicted in Table 1.

**Table 1:** Clinical and demographic parameters of the study population. Mean ± standard deviation values or number and percentages are shown.

Parameter	
Age of recipient (yrs)	57.34 ± 10.37
Age of donor (yrs)	61.65 ± 9.24
Males (%), recipient	78 (65.5)
Females (%), recipient	41 (34.5)
Males (%), donor	54 (63.53)
Females (%), donor	31 (36.47)
Time on dialysis (yrs)	4.22 ± 3.97
History of CV events in recipient	24 (20.2)
History of CV events in donor	18 (20.5)
Weight, recipient (kg)	76.49 ± 15.35
BMI, recipient	28.61 ± 5.13
Hypertension, recipient	96 (80.7)
Hypertension, donor	50 (56.8)
DM, recipient	19 (16)
DM, donor	18 (20.5)
Hyperlipidemia, recipient	52 (43.7)
Hyperlipidemia, donor	18 (20.5)
HLA	
1–3	84 (70.59)
4–5	35 (29.41)
Delayed graft function	43 (36.1)
Acute rejection	18 (15.1)
Graft loss	27 (22.7)
Cold ischemia time (hours)	17.27 ± 19.33
Creatinine serum concentration (mg/dl)	1.68 ± 0.77
eGFR (ml/min) (MDRD)	51.38 ± 17.69

BMI, body mass index; DM, diabetes mellitus; eGFR, estimated glomerular filtration rate.

The most frequent primary kidney diseases in our series were: glomerulonephritis (35.9%), polycystic kidney disease (17.1%) and chronic interstitial nephritis (12.0%). Other conditions accounted for 13.2% of cases. The specific condition could not be determined in 21.8% of the recipients.

## Association of SNPs in the arachidonic pathway and biopsy scores

Table 2 shows the genotype distribution of the six SNPs selected for the study in the *CYP* and *EPHX2* genes. Minor allele frequencies, which ranged from 0.035 to 0.435, did not differ significantly from those expected by the Hardy Weinberg equilibrium (p> 0.05) (Table 2).

**Table 2:** Genotypic and allelic frequencies in the 85 kidney donors.

Polymorphism		N	%	MAF	HWEp
*CYP4F2* V433M	VV	26	30.59	0.435	0.825
	VM	44	51.76		
	MM	15	17.65		
CYP2C8*3	*1/*1	65	76.47	0.128	0.619
	*1/*3	18	21.18		
	*3/*3	2	2.35		
CYP2J2*7	*1/*1	79	92.94	0.035	1
	*1/*7	6	7.06		
	*7/*7	0	0.00		
*EPHX2* K55R	KK	74	87.06	0.065	1
	KR	11	12.94		
	RR	0	0.00		
*EPHX2* R287Q	RR	65	76.47	0.118	0.594
	RQ	20	23.53		
	QQ	0	0.00		
*EPHX2* 3'UTR A>G	AA	41	48.24	0.306	1
	AG	36	42.35		
	GG	8	9.41		

N, number of subjects; MAF, minor allele frequency; HWEp, p-value for Hardy-Weinberg equilibrium

For this analysis, we discarded duplicated kidneys (n = 34), as they obviously had the same polymorphisms and pretransplant scores. The association between the SNPs of the donor and the biopsy score blindly assigned by pathologists to the grafts revealed a statistical trend towards higher scores (more tissue damage) in biopsies from carriers of the CYP2C8*3 variant. Thus, 38% of donors with high scores were carrying the variant, compared with only 17% of carriers among donors with low scores [OR = 2.9 (0.99–8.86), p = 0.052]. The remaining SNPs did not display any significant effect (Table 3).

**Table 3:** Crude analyses for the association between polymorphisms in CYP and *EPHX2* genes of the donor and pretransplant renal biopsy scores.

		Low Score		High Score		OR (CI)	p
		N	%	N	%		
*CYP4F2* V433M	VV	20	31.2	6	28.6	Ref.	
	VM/MM	44	68.8	15	71.4	1.1 (0.38–3.36)	0.524
CYP2C8*3	*1/*1	53	82.8	13	61.9	Ref.	
	*1/*3-*3/*3	11	17.2	8	38.1	2.9 (0.99–8.86)	0.052
CYP2J2*7	*1/*1	59	92.2	20	95.2	Ref.	
	*1/*7-*7/*7	5	7.8	1	4.8	0.59 (0.07–5.36)	0.538
*EPHX2* K55R	KK	56	87.5	18	85.7	Ref.	
	KR/RR	8	12.5	3	14.3	1.17 (0.28–4.87)	0.545
*EPHX2* R287Q	RR	48	75	17	81	Ref.	
	RQ/QQ	16	25	4	19	0.71 (0.21–2.41)	0.407
*EPHX2* 3'UTR A>G	AA	31	48.4	10	47.6	Ref.	
	AG/GG	33	51.6	11	52.4	1.03 (0.39–2.77)	0.574

OR (CI), odds ratio with 95% confidence intervals; Ref., reference.

Next, we included this CYP2C8*3 SNP in a logistic regression model with different clinical and demographic covariates of the donors, with the aim to confirm the association between this variant with the biopsies scores. The results showed that CYP2C8*3 variant was significantly associated with kidney damage when controlling for meaningful covariates [OR = 3.35 (1.03–10.93), p = 0.045] (Table 4).

**Table 4:** Multivariate logistic regression model for the association of CYP2C8*3 in the donor and pretransplant biopsy score.

	B	SE	Wald	OR	CI	p
Donor age	-0.025	0.030	0.689	0.98	0.92–1.03	0.406
Donor hyperlipidemia	0.523	0.722	0.525	1.69	0.41–6.94	0.469
Donor diabetes mellitus	-0.904	0.814	1.235	0.41	0.08–2.00	0.267
Donor sex	-1.178	0.566	4.326	0.31	0.10–0.93	0.038
CYP2C8*3	1.208	0.604	4.002	3.35	1.03–10.93	0.045

B, regression coefficient; SE, standard error; OR, odds ratio; CI, 95% confidence interval

### Association of biopsy scores with clinical outcomes

**Fig 1:**
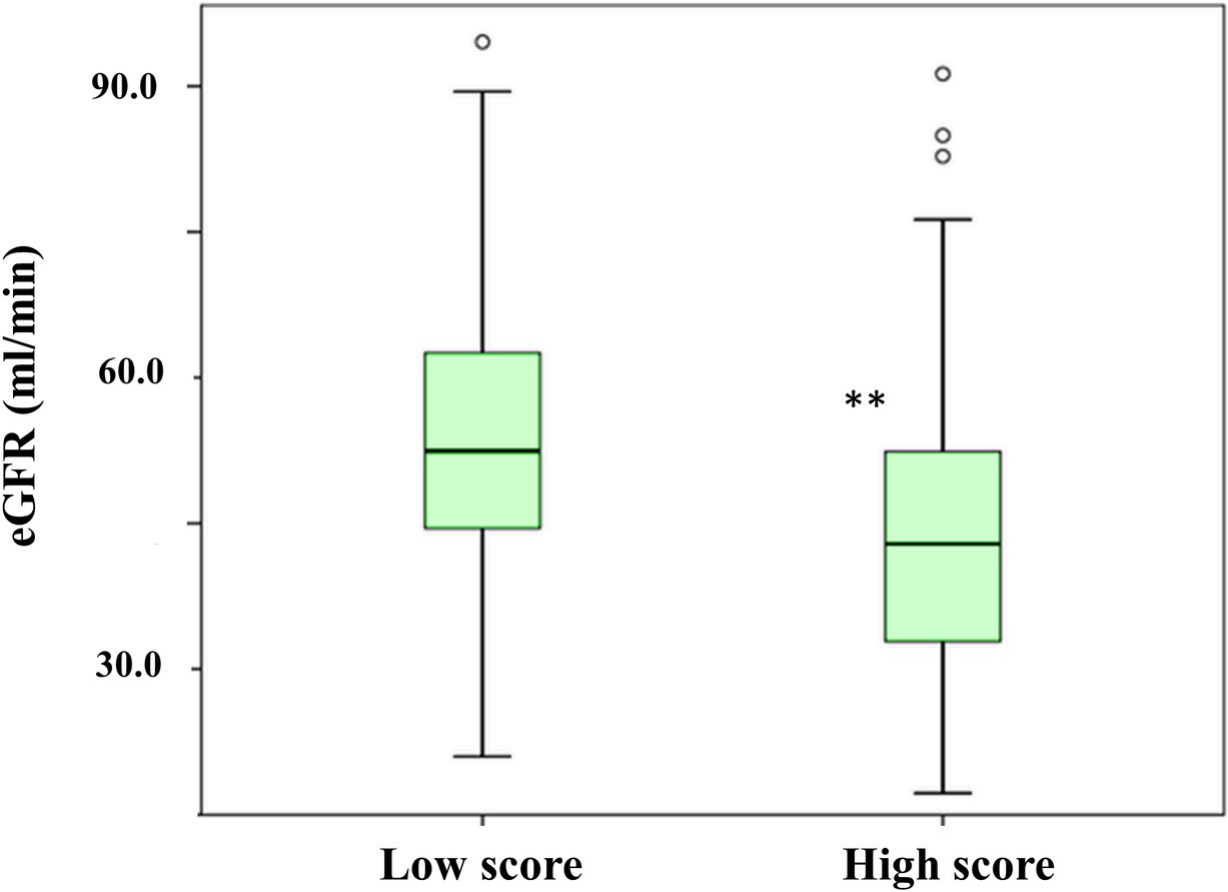
Distribution of estimated glomerular filtration rate (eGFR) values one year after grafting according to the score assigned to the pretransplant biopsies. **p = 0.01.

Out of the 119 grafts analyzed, 88 (73.9%) displayed scores lower than 5, whilst 31 (26.1%) had scores of 5 or higher. In order to assess whether this biopsy score (low vs. high) was predictive of the evolution of the graft in our series of renal transplant recipients, we studied in different logistic regression models the association with both renal function and with the occurrence of acute rejection and delayed graft function in the first year after grafting. Indeed, we observed that patients whose grafts had been assigned higher scores showed significantly worse eGFR (mean values were 45.68 ± 16.05 and 53.04 ± 16.93 ml/min for the high- and low-score group, respectively; p = 0.010) (Fig 1). The association analysis was adjusted by donors’ and recipients’ data, namely age, weight, hypertension and diabetes mellitus, time in dialysis, acute tubular necrosis, cause of donor death and immunosuppressive treatment.

Regarding the association with acute rejection, patients whose donors had higher scores showed significantly higher risk of this complication [OR = 5.28 (1.32–21.13), p = 0.019] (Table 5). The score was however not predictive of DGF [OR = 1.16 (0.50–2.70), p = 0.445]

**Table 5:** Multivariate logistic regression analysis for the association of pretransplant biopsy score with acute rejection.

	B	SE	Wald	OR	CI	p
Pretransplant biopsy score	1.663	0.708	5.520	5.28	1.32–21.13	0.019
Donor age	-0.067	0.038	3.169	0.94	0.87–1.01	0.075
Recipient weight	0.014	0.019	0.530	1.01	0.98–1.05	0.467
Recipient age	0.019	0.040	0.229	1.02	0.94–1.10	0.632
Recipient hypertension	0.642	0.845	0.578	1.90	0.36–9.96	0.447
Recipient hyperlipidemia	-0.724	0.653	1.229	0.48	0.13–1.74	0.268
High HLA mismatch	0.037	0.674	0.003	1.04	0.28–3.89	0.956
Acute tubular necrosis	0.488	0.625	0.610	1.63	0.48–5.55	0.435
Cold ischemia time	-0.076	0.048	2.440	0.93	0.84–1.02	0.118
Time in dialysis	0.102	0.065	2.429	1.11	0.97–1.26	0.119
CV history in recipient	1.032	0.678	2.315	2.81	0.74–10.61	0.128
Tacrolimus vs. cyclosporine	0.958	1.336	0.514	2.61	0.19–35.74	0.473

B, regression coefficient; SE, standard error; OR, odds ratio; CI, 95% confidence interval

### Influence of donor genetics on clinical outcomes

Finally, we studied the possible influence of donor genetic variability in the clinical course of the transplant, again considering acute rejection, delayed graft function and eGFR one year after grafting. Analyses were adjusted by the same clinical and demographic covariates formerly described.

Altered eGFR one year after grafting was observed for recipients with donors that carried *CYP4F2* 433M (S1 Fig). Mean eGFR values for 433M carriers vs. non-carriers were respectively 48.96 ± 16.89 and 55.94 ± 18.62 ml/min (p = 0.038).

In addition, patients whose donors carried the same *CYP4F2* 433M variant showed higher risk of acute rejection. The percentage of carriers in patients with the complication was 88.9%, compared with 63.5% in the group without rejection [OR = 6.18 (1.03–37.21), p = 0.047]. Finally, the CYP2J2*7 variant in the donor was related to higher risk of DGF. Indeed, 17.1% of patients with this complication carried the *7 allele, whilst the variant was present in only 1.4% of patients who did not experience DGF [OR = 25.68 (1.52–43.53), p = 0.025]. The regression models are shown in supplementary S2 and S3 Tables.

**Fig 2:**
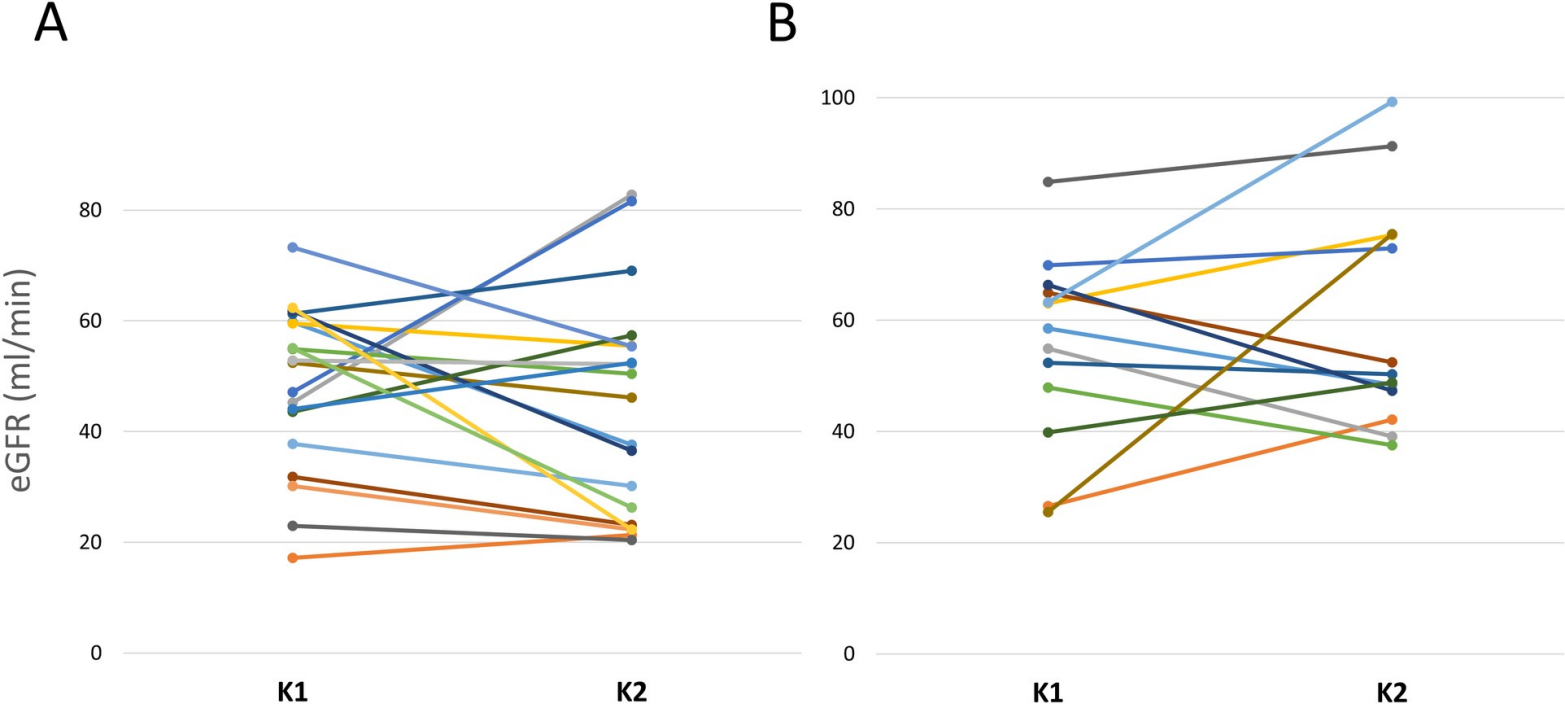
**Estimated glomerular filtration rate (eGFR) for kidney pairs that carried (A) or did not carry (B) the *CYP4F2* 433M variant.** K, kidney.

Finally, we reexamined the observed genetic/clinical associations in a subgroup of 68 of the 119 transplanted kidneys that were paired organs, i.e. from the same donor and therefore carrying the same genetic background. There was no significant differences in the eGFR displayed by each of the kidneys in the paired transplants. This was true either for kidneys that carried the variant (eGFR mean values of kidney 1 vs. kidney 2 = 48.06 ± 14.76 and 44.36 ± 20.03 respectively, p = 0.418) or for those pairs which were non-carriers (55.21 ± 16.96 and 60.02 ± 20.42 ml/min for kidneys 1 and 2 respectively, p = 0.413 (Fig 2). Interestingly, there was a statistically significant difference in the eGFR between pairs that carried the 433M allele and those who did not (mean eGFR = 46.21 ± 17.45 vs. 57.61 ± 18.55 for carriers and non-carriers respectively, p = 0.017)

With regard to the association between the 433M variant and acute rejection in this subgroup, 26.3% of carriers experienced rejection vs. 7.7% of non-carriers (p = 0.057). However, only in one of the kidney pairs we observed the same outcome (rejection), all the remaining pairs had different outcomes. The effect of the CYP2J2*7 in this subgroup could not be reassessed because of the low number of carriers.

## Discussion

A large number of patients with chronic kidney disease depend on a renal transplant to survive. For this reason, a histological evaluation of donor kidney tissue has become an essential aspect in the assessment of renal allograft organ quality, in particular with the increasing use of marginal donors. The identification of genetic biomarkers that determine the predisposition of the donor to have renal damage may therefore be an important tool that adds up to the histological findings to predict how good the graft functionality will be after the transplant. In addition, we hypothesize that the presence of these genetic variants could also correlate with the occurrence of graft-related complications in the recipient.

In the present work, we studied the possible relationship of six common, functional SNPs of the donor in *EPHX2* and several CYP genes with the biopsy score of the graft and with clinical outcomes in the recipient in the first year after grafting. We observed that donor genetic variability may be an important factor influencing kidney damage. Specifically, donors who were carriers of CYP2C8*3 had been blindly assigned higher pretansplant biopsy scores (meaning more tissue damage) that non carriers. The EETs synthesized by *CYP2C8* have renoprotective properties, acting in numerous situations as vasodilator and anti-inflammatory mediators [28]. Therefore, it is reasonable to think that if a donor carries a functional polymorphism in this gene, EETs levels in the kidney (where this enzyme is expressed [29]) will be lower, and therefore the tissue will presumably be more vulnerable to damage. It should be noted, however, that histological data on specific cell types and counts in the biopsies were lacking.

In a previous work by our group [5], we had already observed how the presence of the CYP2C8*3 variant, this time in the recipient, was associated with worse graft function, suggesting systemic low EETs levels resulting in an adverse outcome. Moreover, there are some earlier results by Dai et al. reporting how the CYP2C8*3 variant showed only 15% of the in vitro activity of the wild type allele in the metabolism of arachidonic acid to EETs, which also seems to point in this direction. Indeed, the authors proposed that a defective production of these mediators in organs such as the heart, liver and kidney could lead to pathological changes or disease [13]. Smith et al. also confirmed the in vitro observation that the CYP2C8*3 variant was deficient in the production of EETs (by 75% according to these authors) and, interestingly enough, that this variant in the recipient was associated with kidney damage [14]. Our work is the first study to our knowledge that has extended these implications to the donor´s genotype, which would most likely be a genetic background with more impact on local processes in the graft.

Donor sex was the other factor that showed a significant association with kidney damage, with males showing slightly better pretransplant scores. A study by Sánchez-Escuredo [30] found the opposite association, with males being more frequent among kidneys discarded for transplant. In contrast, other studies have suggested poorer overall graft survival [31] and higher rates of acute rejection [32] for kidneys from female donors independently of the recipient’s sex. Since the impact of donor sex on the outcome of renal transplant is still unclear and because our study design did not primarily aim to establish sex differences, it would be adventurous to draw any conclusions on this matter.

We also examined whether the pretransplant renal score established by the pathologists correlated with the clinical evolution of the recipients. Patients whose grafts had higher biopsy scores had lower eGFR values one year after grafting. This is clinically relevant because previous studies have demonstrated that post-transplant renal function in the first year predicts long‐term kidney transplant survival [33]. Our results are consistent with those obtained by Anglicheau et al, who also observed how in the first twelve months after transplant, renal function was lower in those recipients whose kidneys had been assigned higher pretansplant scores [3]. However, we could not calculate whether the extent of this reduction in renal function was comparable to that reported in our patients (16.35%), as the authors did not show quantitative data of eGFR.

We also observed that those recipients whose grafts had higher scores showed an elevated risk of acute rejection compared with recipients that received kidneys with lower scores. A similar observation has previously been reported by Yilmaz et al [34], who found that higher biopsy scores correlated extremely well with the occurrence of acute rejection episodes during the first year after transplantation. It should be noted, though, that these authors utilized damage scores from protocol biopsies taken one year after the transplant, and hence their results are not easy to compare with those reported herein. Finally, we did not detect a relationship of the biopsy score with the occurrence of DGF in the first year after grafting. Other studies did find such association [35, 36]. The reason for this discrepancy might be our relatively low sample size. Being this primarily a genetic association study, its sample size was limited by the availability of genetic material. As all kidneys were obtained from deceased individuals (living-donor transplants are not being conducted in our hospital yet), the number of donors whose DNA could be isolated was reduced. Furthermore, biopsy scores were not existing for all donors with available DNA.

There are ongoing efforts focused on incorporating genetics to the array of existing clinical and histological parameters that try to predict outcomes in renal transplantation [37, 38]. However, the vast majority of these studies are designed to detect genetic biomarkers in the recipient, in particular in deceased-donor kidney transplantation, where, as we mentioned above, the availability of donor’s genomic DNA is much lower. In this regard, the third part of the present study aimed to investigate the putative correlation between genetic variants in the donor with clinical outcomes one year after grafting. We found that patients whose donors carried the *CYP4F2* 433M variant allele showed worse renal function that non-carriers at that time. In addition, this 433M variant and the CYP2J2*7 SNP were found to increase the risk of acute rejection and delayed graft function, respectively. The results point again to a reduced anti-inflammatory or vasodilator capacity due to an impaired production of EETs and/or to altered production of 20-HETE (synthesized by CYP4F2) in the grafts that carried these variants. There are a few studies, including some conducted by our group in an independent series of renal transplant recipients, [10–12, 39, 40] that also show how the presence of genetic alterations in the donor may influence graft function and the risk of renal transplant complications. In any case, the role of the genes involved in the metabolism of arachidonic acid to EETs and 20-HETE in the donor has been virtually unexplored to date.

We also aimed to evaluate how influential the recipients’ characteristics were when donor genetics are out of the equation. For this, we re-analyzed the observed clinical associations in a subgroup of paired kidneys (same donor and therefore same genetics). Fig 2 shows that indeed renal function was not significantly different among paired organs who were carriers of the CYP4F2 433M variant. Differences were however visible when kidney pairs carrying and not carrying the allele were compared. In contrast, the association with acute rejection showed a different profile, as the kidney pairs did not mostly share the same outcome. It is tempting to speculate that recipient characteristics, e.g. immunological, have a bigger role in this case.

The main limitation of this study was its relatively low sample size, which was a consequence of the above-mentioned difficulties to obtain grafts with both histological data and availability of genetic material. This resulted in some wide confidence intervals for the analysis of associations between clinical outcomes and low-frequency SNPs. In addition, being this a retrospective study, we could not measure EETs or 20-HETE plasma/urine concentrations in our patients, which could have help elucidate the mechanisms underlying our observations. In this regard, our group is currently developing a project to measure these concentrations and examine their correlation with donors’ and recipients’ genetic polymorphisms. Finally, the analysis of other relevant genes in this route, e.g. *CYP4A11*, could have also provided with useful information.

In summary, our findings show, for the first time to our knowledge, that SNPs in donor genes that are involved in the synthesis of vasoactive eicosanoids may contribute to increased graft damage in kidney transplantation. Furthermore, we observed that this increased damage correlated with worse graft function and outcomes in the recipients. Finally, we could also confirm that donor genetic variations may also be independent risk factors for clinical outcomes in patients receiving a kidney transplant. All these results taken together indicate that the addition of genetic testing in the donor prior to transplantation could improve the predictive power of the existing histological and clinical parameters regarding the clinical evolution of the recipient. Nevertheless, further studies with larger series of patients are warranted to corroborate the preliminary findings described herein.

## Supporting information

S1 Fig Estimated glomerular filtration rate (eGFR) for carriers and non-carriers of the CYP4F2 433M variant allele. *p = 0.038.
(TIF)

S1 Table List of clinical and demographic variables considered in the study. BMI, body-mass index; CV, cardiovascular; eGFR, estimated glomerular filtration rate.
(DOCX)

S2 Table Multivariate logistic regression analysis for the association of the CYP4F2 433M variant in the donor with acute rejection in renal transplant recipients. B, regression coefficient; SE, standard error; OR, odds ratio; CI, 95% confidence interval
(DOCX)

S3 Table Multivariate logistic regression analysis for the association of the CYP2J2*7 variant in the donor with delayed graft function in renal transplant recipients. B, regression coefficient; SE, standard error; OR, odds ratio; CI, 95% confidence interval; DM, diabetes mellitus.
(DOCX)
