# Supplementary figures and images for: Polymorphisms in vasoactive eicosanoid genes of kidney donors affect biopsy scores and clinical outcomes in renal transplantation
Source: PLoS One. 2019 Oct 17;14(10):e0224129. doi: 10.1371/journal.pone.0224129 (PMC6797116; doi:10.1371/journal.pone.0224129)

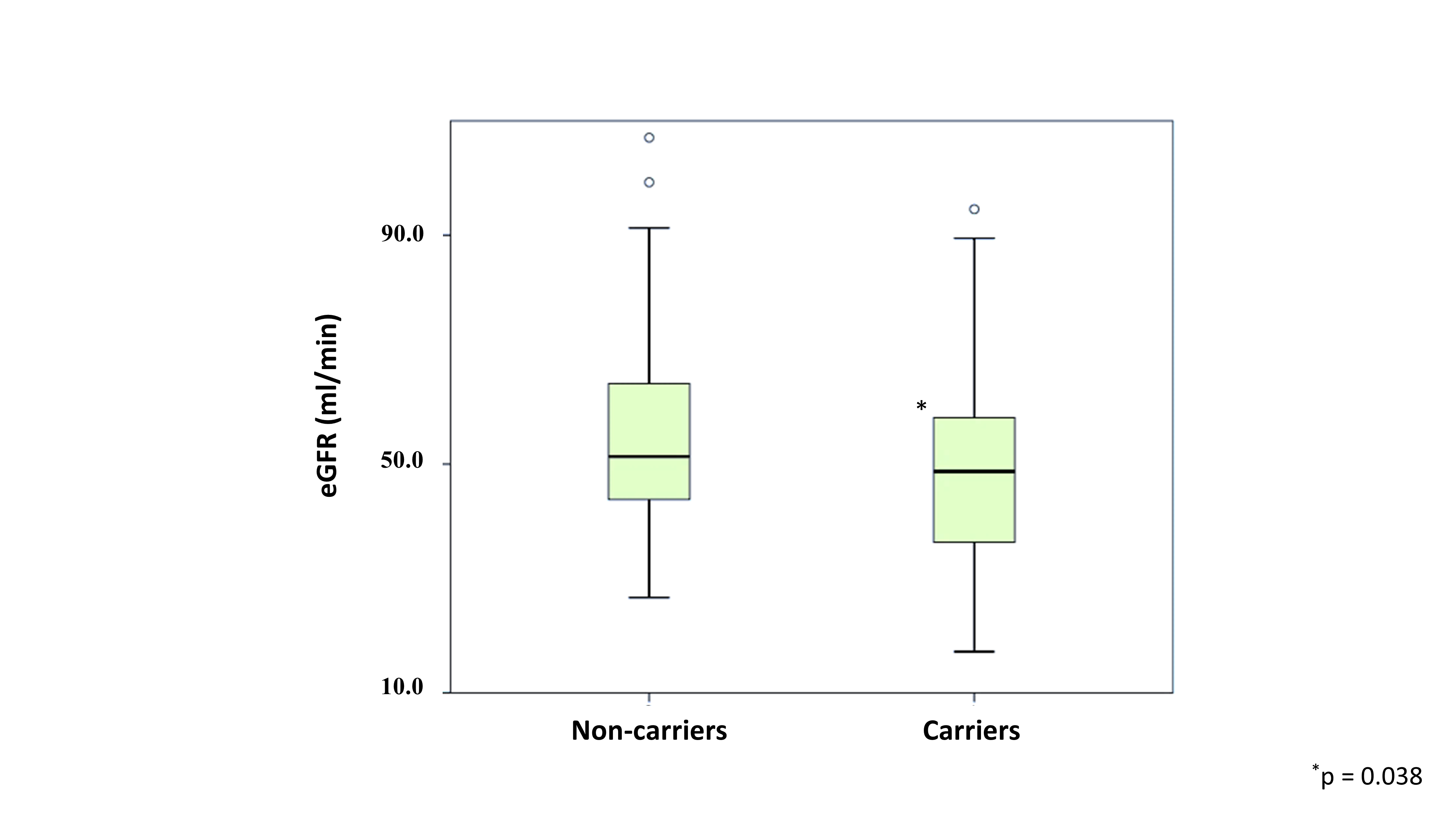

Supplement: S1 Fig — *p = 0.038. (TIF) [file pone.0224129.s001.tif]
