# Supplementary material for: Polymorphisms in vasoactive eicosanoid genes of kidney donors affect biopsy scores and clinical outcomes in renal transplantation
Source: PLoS One. 2019 Oct 17;14(10):e0224129. doi: 10.1371/journal.pone.0224129 (PMC6797116; doi:10.1371/journal.pone.0224129)
Supplement: S1 Table — BMI, body-mass index; CV, cardiovascular; eGFR, estimated glomerular filtration rate. (DOCX) [file pone.0224129.s002.docx]

**Supplementary S1 Table. List of clinical and demographic variables considered in the study.**

| **DONOR-RELATED** | **RECIPIENT-RELATED** | **PROCEDURE-RELATED** |
| --- | --- | --- |
| Age | Age | Cold ischemia time |
| Sex | Sex | Graft revascularization time |
| Weight | Weight | HLA mismatch |
| Height | Height | Cyclosporine / Tacrolimus |
| BMI | BMI | Concentration of immunosuppressant |
| CV history | CV history | Use of anti-Il2 receptor antibodies |
| Hypertension | Hypertension |  |
| Diabetes | Diabetes |  |
| Hyperlipidemia | Hyperlipidemia |  |
| Smoking | Smoking |  |
| Cause of death | Time on dialysis |  |
| Genetic variants | Type of dialysis |  |
| Biopsy pretransplant score | Primary renal disease |  |
|  | Hepatitis C virus infection |  |
|  | Cytomegalovirus infection |  |
|  | Renal function (eGFR) |  |
|  | Delayed graft function |  |
|  | Acute rejection |  |
|  | Graft loss |  |
