# Supplementary material for: Polymorphisms in vasoactive eicosanoid genes of kidney donors affect biopsy scores and clinical outcomes in renal transplantation
Source: PLoS One. 2019 Oct 17;14(10):e0224129. doi: 10.1371/journal.pone.0224129 (PMC6797116; doi:10.1371/journal.pone.0224129)
Supplement: S2 Table — B, regression coefficient; SE, standard error; OR, odds ratio; CI, 95% confidence interval (DOCX) [file pone.0224129.s003.docx]

**Supplementary S2 Table. Multivariate logistic regression analysis for the association of the *CYP4F2* 433M variant in the donor with acute rejection in renal transplant recipients**

|  | **B** | **SE** | **Wald** | **OR** | **CI** | **p** |
| --- | --- | --- | --- | --- | --- | --- |
| *CYP4F2 V433M* | 1.821 | 0.916 | 3.952 | 6.18 | 1.03-37.21 | 0.047 |
| Donor age | -0.077 | 0.039 | 3.894 | 0.93 | 0.86-1.00 | 0.048 |
| Recipient weight | 0.022 | 0.020 | 1.218 | 1.02 | 0.98-1.06 | 0.270 |
| Recipient age | 0.017 | 0.035 | 0.224 | 1.02 | 0.95-1.09 | 0.636 |
| Recipient hypertension | 0.371 | 0.867 | 0.183 | 1.45 | 0.26-7.92 | 0.669 |
| Recipient hyperlipidemia | -0.382 | 0.666 | 0.329 | 0.68 | 0.19-2.52 | 0.566 |
| High HLA mismatch | -0.065 | 0.695 | 0.009 | 0.94 | 0.24-3.66 | 0.925 |
| Acute tubular necrosis | 0.442 | 0.650 | 0.463 | 1.56 | 0,44-5.56 | 0.496 |
| Cold ischemia time | -0.041 | 0.047 | 0.776 | 0.96 | 0,88-1.05 | 0.378 |
| Time in dialysis | 0.066 | 0.066 | 1.001 | 1.07 | 0.94-1.22 | 0.317 |
| CV history, recipient | 0.716 | 0.670 | 1.144 | 2.05 | 0.55-7.61 | 0.285 |
| Tacrolimus vs. cyclosporine | 0.912 | 1.311 | 0.484 | 2.49 | 0.19-32.49 | 0.487 |

B, regression coefficient; SE, standard error; OR, odds ratio; CI, 95% confidence interval
