# Supplementary material for: Polymorphisms in vasoactive eicosanoid genes of kidney donors affect biopsy scores and clinical outcomes in renal transplantation
Source: PLoS One. 2019 Oct 17;14(10):e0224129. doi: 10.1371/journal.pone.0224129 (PMC6797116; doi:10.1371/journal.pone.0224129)
Supplement: S3 Table — B, regression coefficient; SE, standard error; OR, odds ratio; CI, 95% confidence interval; DM, diabetes mellitus. (DOCX) [file pone.0224129.s004.docx]

**Supplementary S3 Table. Multivariate logistic regression analysis for the association of the *CYP2J2*7* variant in the donor with delayed graft function in renal transplant recipients.**

|  | **B** | **SE** | **Wald** | **p** | **OR** | **CI** |
| --- | --- | --- | --- | --- | --- | --- |
| *CYP2J2*7* | 3.246 | 1.444 | 5.052 | 0.025 | 25.68 | 1.52-43.53 |
| Age of recipient | -0.008 | 0.027 | 0.095 | 0.758 | 0.99 | 0.94-1.05 |
| Hypertension, recipient | 0.148 | 0.647 | 0.053 | 0.819 | 1.16 | 0.33-4.12 |
| Age of donor | -0.016 | 0.034 | 0.225 | 0.635 | 0.98 | 0.92-1.05 |
| Time in dialysis | 0.133 | 0.068 | 3.763 | 0.052 | 1.14 | 1.00-1.31 |
| eGFR | -0.041 | 0.018 | 4.832 | 0.028 | 0.96 | 0.93-1.00 |
| DM, recipient | -0.914 | 0.773 | 1.399 | 0.237 | 0.40 | 0.09-1.82 |
| Hypertension, donor | -0.312 | 0.548 | 0.325 | 0.569 | 0.73 | 0.25-2.14 |
| DM, donor | -0.245 | 0.657 | 0.139 | 0.709 | 0.78 | 0.22-2.84 |
| Weigh, recipient | 0.041 | 0.019 | 4.473 | 0.034 | 1.04 | 1.00-1.08 |
| Cause of donor death | 0.616 | 0.928 | 0.440 | 0.507 | 1.85 | 0.30-11.42 |
| Tacrolimus vs. cyclosporine | -1.166 | 1.290 | 0.817 | 0.366 | 0.31 | 0.02-3.90 |

B, regression coefficient; SE, standard error; OR, odds ratio; CI, 95% confidence interval; DM, diabetes mellitus
